# An integrated numerical modelling framework for simulation of the multiphysics in sonoprocessing of materials^☆^

**DOI:** 10.1016/j.ultsonch.2025.107428

**Published:** 2025-06-13

**Authors:** Ling Qin, Kang Xiang, Lianxia Li, Iakovos Tzanakis, Dmitry Eskin, Jiawei Mi

**Affiliations:** a School of Engineering, University of Hull, East Yorkshire, HU6 7RX, UK; b Centre of Innovation for Flow Through Porous Media, Department of Petroleum Engineering, University of Wyoming, Laramie, WY 82071, USA; c Departments of Mathematics, University of North Carolina at Chapel Hill, NC 27514, USA; d Department of Mechanical Engineering and Mathematical Sciences, Oxford Brookes University, Oxford OX3 0BP, UK; e Brunel Centre for Advanced Solidification Technology, Brunel University of London, Uxbridge UB8 3PH, UK

**Keywords:** Multiphysics modelling, Sonoprocessing, Bubble dynamics and implosion, Shockwave, Wave-structure interaction

## Abstract

We have designed, developed, and integrated a comprehensive mathematical and numerical modelling framework for simulations of the complex physics and highly dynamic phenomena that occur across different length and time scales in the processes of sonochemistry and sonication of materials. The framework comprises three interconnected sub-models: (1) a bubble oscillation and implosion model, (2) a shock wave emission and propagation model, and (3) a wave–structure interaction (WSI) model. Firstly, we described in detail the governing equations, numerical schemes, boundary and initial conditions used in each sub-model with a particular emphasis on the data mapping methods for numerically linking the three sub-models together. Then, we present a number of simulation cases to demonstrate the power and usefulness of the model. We also did systematic model validation and calibration using the *in-situ* and real-time collected big X-ray image data. This is the first time such comprehensive and high-fidelity numerical models have been achieved for sonoprocessing of materials. Complementary to the most advanced *in-situ* and operando experiments, the integrated model is an indispensable modelling tool for computational studies and optimizations of the ultrasound-assisted chemical synthesis and sonoprocessing of materials.

## 1 Introduction

In a continuous liquid flow containing micrometre sized gaseous and/or solid phases (e.g., bubbles and/or particles), complex multiphysics interactions often occur among these phases at different length (from nm to mm) and time scales (from ns to mins) [1,2]. These are often found in natural water systems, i.e., in rivers, lakes, and oceans, and in a wide range of man-made physical or biomedical systems [[3], [4], [5], [6]]. Among those interactions, the creation, growth, oscillation, transportation and annihilation of the cavitation bubbles due to the local pressure variation within the liquid media have attracted vast amount of interests in many scientific disciplines and industrial areas since the first experimental observation made by Knapp *et al.* in 1948 [7]. Until now, the most intriguing phenomenon that is still attracting intense research is to understand the implosion dynamics of cavitation bubbles in different conditions, the local shockwaves produced by such implosion, and the damaging or beneficial effects [9] of such local shockwaves in a vast number of fields and applications. For example, using pulsed laser-induced cavitation shock waves to target biological tissues[8,9], lithotripsy[10,11], surface cleaning[12,13], textile processing[14,15], etc.

In recent years, ultrasound assisted (or enhanced) chemical synthesis and/or sonoprocessing of structural and functional materials have been rapidly developed. Most processes explore the beneficial effects of the alternative ultrasonic pressure produced cavitation bubbles and the shockwave energy released at the bubble implosion to accelerate chemical reactions, enhance catalytic performance, or enable highly efficient structure fragmentation or layer exfoliation, such as in the case of ultrasound-assisted liquid-phase exfoliation (ULPE) of 2D materials [16,17]. Due to the highly transient nature of μm sized bubble implosion (at ns time scale), especially when occurred in an opaque liquid medium, it is a great challenging endeavour to perform any sensible and meaningful experimental observations. Hence numerical modelling and simulation play a crucial and indispensable role in the research. Most previous modelling activities had done simulations on a single and two bubble systems, which provided valuable insights into the fundamental mechanisms governing the bubble–bubble and bubble–fluid interactions [[18], [19], [20]]. However, most of the bubble data collected for model validation were from the experiments of focused laser pulses induced bubble rather than from those by acoustic excitation[21]. Using laser pulses, precise control can be achieved at bubble nucleation, facilitating the observation of microjet formation, shock wave propagation, and secondary cavitation events [22]. However, the underlying mechanisms of laser-induced bubble nucleation and oscillation are different to those produced in ultrasonic pressure fields. The cyclic acoustic pressure fields produce and drive much larger number of bubbles to be nucleated and imploded stochastically and collectively, forming highly dynamic bubble clouds (containing hundreds of or even thousands of bubbles). Within the bubble cloud region, there are highly frequent multiple bubble interactions, e.g., bubble distortion, collision, coalescence, shielding, asymmetric collapse, etc. These are not typically observed in the laser-induced single-bubble and the associated modelling [6,23,24, 25]. Hence, the inherently chaotic and bubble cloud collective behaviour are the main characteristics often found in sonoprocessing, which have not been systematically addressed by numerical modelling. In addition, many other complexities (i.e., multiphysics phenomena) exist inherently in sonoprocessing of different materials, e.g., rapid changes of temperature, differences in density, viscosity and surface tension, etc. The multiphysics and integrated numerical model reported here is purposely developed to take those issues into account in the simulation.

In our earlier work, we developed a numerical model that combines the Volume of Fluid (VOF) and Continuum Surface Force (CSF) methods to simulate the growth, oscillation, and implosion of a single bubble in ultrasound fields. This model was experimentally validated using synchrotron X-ray imaging data [16]. Here, we further extended the model to deal with the dynamics of multiple bubbles, including the generation, propagation, and interaction of shock waves, as well as their effects on bulk materials in viscous fluids. Furthermore, we used a large amount of *in-situ* and real-time collected X-ray imaging data for the model validation and calibration, achieved high fidelity modelling of the complex physics in the sonoprocessing of materials.

## 2 Mathematical formulation and numerical methods

### 2.1 Governing equations for modelling bubble oscillation and implosion

The mathematical formula and governing equations for modelling bubble oscillation and implosion are listed below. Eq. (1) and Eq. (2) are the continuity equations for the liquid and gas phase (bubble) respectively [25]:

(1) $$\frac{\partial (\alpha_{l}\rho_{l})}{\partial t}+\nabla ∙\left(\alpha_{l}\rho_{l}U\right)\;=0$$

(2) $$\frac{\partial (\alpha_{g}\rho_{g})}{\partial t}+\nabla ∙\left(\alpha_{g}\rho_{g}U\right)=0$$

where $\rho_{l}$ and $\rho_{g}$ are the liquid and gas densities respectively; $\alpha_{l}$ and $\alpha_{g}$ are the volume fractions and $\alpha_{l}+\alpha_{g}=1$ with $\alpha_{l}=1$ denoting the liquid, $\alpha_{g}=1$ denoting the bubble; U is the averaged velocity of the two-phase flow. In this model, the bubble and liquid are treated as immiscible phases with no slip between them. The pressure and velocity are shared by both phases. The density and viscosity are averaged based on the volume fraction of each phase.

The VOF is a computationally efficient method for tracking free surfaces [26], hence was chosen here to track the bubble–liquid interfaces. $\alpha_{l}$ is the volume fraction of liquid, $\alpha_{g}=1-\alpha_{l}$ is that of the bubble. α varies from 0 to 1 across the interface region.

The summation of Eq. (1) and Eq. (2) produces the overall continuity equation [27]:

(3) $$\frac{\partial \rho }{\partial t}+\nabla \cdot \left(\rho U\right)=0$$

where $\rho =\rho_{l}\alpha_{l}+\rho_{g}\alpha_{g}$ is the mixed density of the gas and liquid phases.

The momentum equation reads[28]:

(4) $$\frac{\partial }{\partial t}\left(\rho U\right)+\nabla \cdot \left(\rho UU\right)=-\nabla p+\nabla \cdot \tau +\rho g+\sigma k\nabla \alpha_{l}+F_{a}$$

where p is the pressure; g is the acceleration of gravity; σ is the surface tension coefficient; $F_{a}$ is the pressure (force) at the acoustic pressure emitting surface and is calculated by Eq. (12). The term, $\sigma k\nabla \alpha_{l}$, on the right hand of Eq. (4) accounts for the surface tension force acting on the bubble–liquid interface calculated by the CSF method [29,30]; k is the interface curvature calculated by:

(5) $$k=-\nabla \cdot \left(\frac{{\tilde{\alpha }}_{l}}{\left|{\tilde{\alpha }}_{l}\right|}\right)$$

where ${\tilde{\alpha }}_{l}$ is obtained from the volume fraction $\alpha_{l}$ by smoothing it over a finite region along the interface using the Lafaurie filter[31]. $\left|{\tilde{\alpha }}_{l}\right|$ is the absolute value of ${\tilde{\alpha }}_{l}$. More detailed descriptions of the momentum equation are given by Yin *et al.* [32], τ is the viscous stress tensor of a Newtonian fluid and calculated by:

(6) $$\tau =\mu \left(\nabla U+{\left(\nabla U\right)}^{T}-\frac{2}{3}\left(\nabla \cdot U\right)I\right)$$

where I is the unit tensor,$\mu =\mu_{l}\alpha_{l}+\mu_{g}\alpha_{g}$ is the average dynamic viscosity.

The energy equation expressed in terms of temperature T is written as:

$$\left[\frac{\partial \left(\rho T\right)}{\partial t}+\nabla \cdot \left(\rho TU\right)\right]+\left(\frac{\alpha_{l}}{\Omega_{l}}+\frac{\alpha_{g}}{\Omega_{g}}\right)\left[\frac{\partial \left(\rho K\right)}{\partial t}+\nabla \cdot \left(\rho KU\right)\right]$$

(7) $$=\left(\frac{\alpha_{l}}{\Omega_{l}}+\frac{\alpha_{g}}{\Omega_{g}}\right)\left[\frac{\partial p}{\partial t}+\nabla \cdot \left(\tau \cdot U\right)\right]+\left(\frac{\alpha_{l}\lambda_{l}}{\Omega_{l}}+\frac{\alpha_{g}\lambda_{g}}{\Omega_{g}}\right)\left(\nabla^{2}T\right)$$

where $\Omega_{l}$ and $\Omega_{g}$ are the heat capacity of the liquid and gas phases respectively at a constant pressure; $K=U^{2}/2$ is the kinetic energy; ∇·(τ·U) is the shear stress on the flow [33]; $\lambda_{l}$ and $\lambda_{g}$ is the thermal conductivity of the liquid and gas phase, respectively.

For the liquid phase, the Tait equation of state was used [34]:

(8) $$p=\frac{\rho_{0}c_{l}^{2}}{n}\left({\left(\frac{\rho }{\rho_{l}}\right)}^{n}-1\right)+p_{0}$$

where, $\rho_{0}$ = 998.2 kg/m^3^ is liquid (water) density at the reference pressure, $p_{0}$ = 3490 Pa. $c_{l}$ the speed of sound in liquid; the exponent n = 7.15 was used because the deionized water (DIW) has weak compressibility [35]. For the gas phase, a polytropic equation of state was used:

(9) $$p=\chi \rho_{g}^{\gamma }$$

where χ = 0.12  kg/m^3^ is a constant calculated for an ideal gas at 298 K and ambient pressure (10320 Pa) [36]; the exponent γ is dependent on the thermodynamic process inside the bubble. In an isothermal process, it is unity. In our case, γ = 1.04. The justification of using those data can be found in our earlier work [37].

**Fig. 1:**
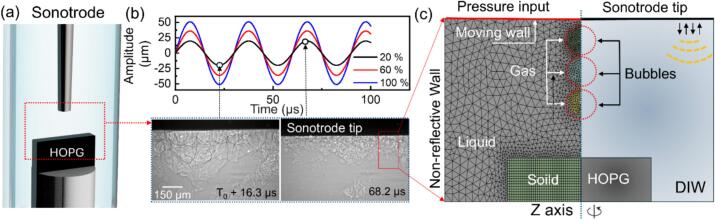
The geometry, mesh structures and boundary conditions used in the bubble dynamics simulations: (a) A schematic of the experimental setup of the ULPE; (b) Measurement of the sonotrode tip vibration peak-to-peak amplitude in the DIW by X-ray radiographic images, which is applied as the boundary condition of the sonotrode tip in (c); (c) The finite volume meshes used for modeling the oscillation and implosion dynamics of three bubbles.

Fig. 1(a) shows the typical sonotrode and sample arrangement inside the deionised water (DIW) contained within a quartz tube holder during the ULPE process, and Fig. 1c presents the mesh and boundary conditions for simulating the oscillation and implosion of three bubbles (an axis-symmetry model). The quartz tube walls were set as non-reflective boundaries. The surface of the sonotrode tip in Fig. 1c was defined as a moving wall, vibrating with a velocity of:

(10) $$V\left(t,y\right)=V_{0}sin\left(\omega t\right)cos\left(\varepsilon y\right)$$

Here, $V_{0}$ is defined as:

(11) $$V_{0}=\frac{p_{a}}{\rho_{l}c_{0}}$$

where $p_{a}$ is the pressure amplitude of the ultrasound applied, $c_{0}$ is speed of sound in the liquid, ω=2πf is the angular frequency and f is frequency of the ultrasound (a Hielscher UP 100H ultrasound processor in this case). In this work, a fixed frequency of 30 kHz was used. $\varepsilon =\omega /c_{l}$ is the wave number of the ultrasound. The peak-to-peak amplitude of sonotrode tip was measured from the X-ray images (see Fig. 1b). To calculate the acoustic pressure $p_{a}$, in our study, vibration amplitudes corresponding to 20 %, 60 %, and 100 % of the full peak-to-peak amplitude *A* (i.e., 39, 72, and 102  μm, respectively) were used. The corresponding acoustic pressure was then obtained as:

(12) $$p_{a}=A\rho c_{l}\omega$$

where ρ, $c_{l}$, and ω denote the liquid density, speed of sound, and angular frequency, respectively.

Furthermore, $F_{a}$ described in Eq.(4) is the mean force per unit volume due to the ultrasound wave. In this case, $F_{a}$ is defined as:

(13) $$F_{a}=\frac{p_{a}^{2}}{\rho_{l}c_{l}^{2}}sin\left(\frac{1}{2}-\cos(\omega t)\right)\left(\varepsilon \sin(2\varepsilon y)\right)$$

where y is the vertical distance away from the wave source in the y-axis direction. Eq. (13) was included in the moving wall boundary condition by using a User-Defined Function (UDF). The liquid properties are listed in Table 1.

**Table 1:** Properties of liquid used for simulation [23,42].

Parameters	Symbol & unit	DIW	Silicone oil (50 cSt)	Silicone oil (1000 cSt)
Sound speed	$C_{0}\left(m.s^{-1}\right)$	1482	987	931
Surface tension	$\sigma (mN.m^{-1})$	72.8	21	25
Dynamic viscosity	μ(cSt)	1	50	1000
Density	$\rho (kg.m^{-3})$	998.2	960	970
Thermal conductivity	λ(W/(m.K))	0.606	0.15	0.13

* All measurements were conducted at room temperature (around 25 °C).

For simulation of three bubbles, a steady-state pressure field without the bubbles was first calculated, then the patch method [38] was used to “insert” the three bubbles into the computational domain.

### 2.2 Governing equation for modelling shockwave propagation

The governing equations for simulating shockwave propagation are listed below. It uses a density-based compressible flow solver based on the central-upwind schemes of Kurganov and Tadmor [39].

The continuity equation is:

(14) $$\frac{\partial p}{\partial t}+\frac{\partial }{\partial x_{i}}\left(u_{i}\rho \right)=0$$

The momentum equation is:

(15) $${\left(\frac{\partial \hat{u_{i}}}{\partial t}\right)}_{I}+\frac{\partial }{\partial x_{j}}\left(u_{i}\hat{u_{i}}\right)+\frac{\partial p}{\partial x_{i}}=0$$

The energy equation is:

(16) $${\left(\frac{\partial \hat{E}}{\partial t}\right)}_{I}+\frac{\partial }{\partial x_{k}}\left[u_{k}\left(\hat{E}+p\right)\right]-\frac{\partial }{\partial x_{i}}\mu u_{j}\left(\frac{\partial u_{j}}{\partial x_{i}}+\frac{\partial u_{i}}{\partial x_{i}}-\frac{2}{3}\frac{\partial u_{k}}{\partial x_{k}}\delta_{\mathrm{ij}}\right)=0$$

From $\hat{E}$, the temperature is calculated by

(17) $$T=\frac{1}{{∁}_{v}}\left(\frac{\hat{E}}{\rho }-\frac{u_{k}u_{k}}{2}\right)$$

where, T is temperature, t time,ρ density, μ dynamic viscosity, δ Kronecker delta, $u_{i}$ velocity vector, k turbulent kinetic energy, x space variable, E energy and C gas concentration.

### 2.3 Boundary and initial conditions

#### 2.3.1 Initial conditions

**Fig. 2:**
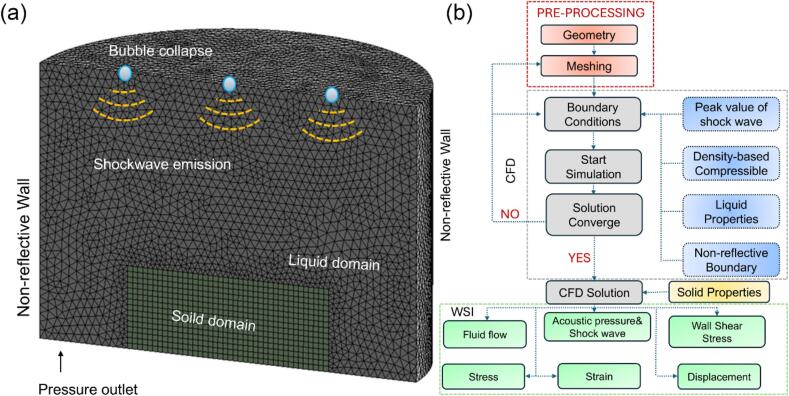
(a) The finite volume mesh structures and boundary conditions used in simulating propagation of the shockwaves produced by multi-bubble implosion; (b) A flowchart illustrating the procedures and coupling between CFD and WSI simulations.

At the instant of bubble implosion, shockwaves were emitted from the bubble center. Based on the previously established bubble dynamics model in section 2.1, the initial shockwave intensity was determined. Then, the patch method was used again to define the bubble center as the origin of the shockwaves (see Fig. 2a) as the initial condition, allowing them to propagate 1 mm before reaching the surface of the bulk material.

#### 2.3.2 Wall boundary condition

For the flow at the wall, the boundary condition was set as non-slip and non-reflective wall. When a shock wave impacts onto a solid boundary, full or partial wave reflection may occur, causing complex interference patterns such as standing waves or secondary shock waves [40]. However, in the current model, we only simulated the first impact of the shock wave without considering any subsequent wave reflection [16,41], hence, non-reflective boundary conditions were used here.

Hence, the velocity at the wall is

(18) $$\rho u_{i}=0$$

The pressure at the wall is:

(19) $$\frac{\partial p}{\partial n}=0$$

The temperature at the wall is (assuming an adiabatic condition):

(20) $$\frac{\partial T}{\partial n}=0$$

Assuming an ideal gas, the state equation is:

(21) $$\frac{\partial \hat{E}}{\partial n}=0$$

where n is the normal direction to the wall.

(3) Outlet boundary condition

The outlet boundary is defined as a pressure outlet, where the pressure is set equal to the atmospheric pressure.

(22) $$p_{\mathrm{outlet}}=p_{\mathrm{ambient}}$$

All gradients of other parameters are assumed to be zero.

### 2.4 Model and governing equations for wave- structure interaction

The Finite Element Analysis (FEA) based WSI model in ANSYS Mechanical was used to simulate the forces and stresses induced by the shockwaves onto the bulk material (the Highly oriented pyrolytic graphite, HOPG in this study).

The governing equations for an anisotropic, linear-elastic solid are [43]:

(23) $$\left[M_{s}\right]\left\{\ddot{\nu }\right\}+\left[N_{s}\right]\left\{\nu \right\}=\left[F_{s}\right]+\left[\varphi \right]\left\{p\right\}$$

(24) $$\left[\begin{array}{c} M_{s}\;\text{0}\;\\ \rho \varphi^{T}\;{\text{M}}_{f} \end{array}\right]\left\{\frac{\ddot{\nu }}{\ddot{p}}\right\}+\left[\begin{array}{c} N_{s}\;\varphi \;\\ 0\;{\text{N}}_{f} \end{array}\right]\left\{\frac{\nu }{p}\right\}=\frac{F_{s}}{F_{f}}$$

where $\left\{\nu \right\}$ is the nodal displacement vector and $\left\{\ddot{\nu }\right\}$ is the acceleration vector. $\left[M_{s}\right]$ is the structural mass matrix; $\left[M_{f}\right]$ is the fluid mass matrix $.\left[N_{s}\right]$ and $\left[N_{f}\right]$ are the structural and fluid stiffness matrix; $\left[F_{s}\right]$ and $\left[F_{f}\right]$ are the structural and fluid force matrix.$\left[\varphi \right]$ is a coupling matrix that represents the effective surface area associated with each node in the wave-structure interface.

In the WSI modelling, the pressure, p, produced by the shockwave induced by bubble implosion was taken as the load boundary conditions in Eqs. (23), (24), following a typical one-way coupling strategy commonly used in Fig. 2b. Then the $\left\{\nu \right\}$ and $\left\{\ddot{\nu }\right\}$ of the HOPG sheet was calculated. The properties of HOPG used in the WSI simulation are listed in Table 2.

**Table 2:** Properties used for WSI simulation [44,45].

Parameters	Symbol & unit	HOPG
Compressive strength	$\sigma_{c}$(MPa)	100
Tensile strength	$\sigma_{t}$(MPa)	25
Young's modulus	G(GPa)	20
Density	$\rho (g/{\mathrm{cm}}^{3})$	2.26

* All the data of HOPG were measured along the interlayer direction.

### 2.5 Numerical method and computing hardware

For bubble dynamics modelling, the SIMPLE algorithm [46] was used for density–velocity coupling in Eq. (1), (2). For the shockwave propagation, Eq. (14), (15) were solved using the Pressure Implicit with Splitting of Operators (PISO) algorithm [47]. All simulations were performed in double precision using a segregated solver, with the time steps ranging from 1 × 10^−7^ s to 1 × 10^−10^ s. The bubble dynamics simulations were performed using the open-source CFD platform OpenFOAM v2412. Simulations involving shockwave propagation and its coupling with solid media were conducted using ANSYS Fluent 2024R2 and ANSYS Mechanical 2024, respectively. The data between Fluent and ANSYS Mechanical were directly transferred via ANSYS Workbench. All numerical simulations were carried out on an HP Workstation Z2 G9. Each CFD simulation case required approximately 83 computational hours, while each WSI simulation case took approximately 32 h to complete.

### 2.6 Imaging data from *in-situ* experiments for model calibration and validation

Since 2011, by exploiting the unique advantages of the ultrafast and high-speed synchrotron X-ray imaging capabilities available at 32-ID of the Advanced Photon Source, Argonne National Laboratory (USA), and I12 of the Diamond Light Source (UK), and the ID19 of the ESRF, researchers in Mi’s group have conducted systematic studies on the highly dynamic behavior of ultrasonic bubble oscillation, implosion, and shock wave propagation in different liquid media, including high-temperature liquid metals [[48], [49], [50]]. Earlier studies primarily focused on validating bubble oscillations and the associated interfacial instabilities [37]. Here, we focus on elucidating the effects of viscosity on ultrasonic bubble dynamics. A series of *in-situ* optical and x-ray images were presented and compared with the modelled results.

## 3 Case studies, comparation with experiments and previous modelling work

**Fig. 3:**
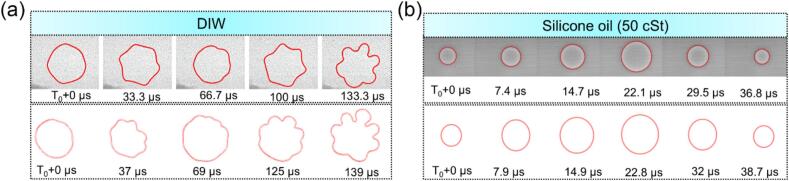
Top row: the ultrafast synchrotron X-ray images of the oscillating bubbles captured at the sector 32-ID-B of the Advanced Photon Source in (a) DIW and (b) silicone oil of 50 cst. Bottom row: the corresponding simulation results for comparison. (More dynamic information is illustrated in Video 1).

A series of high-speed optical and X-ray images are presented here and compared with the simulations with the focus on elucidating the effects of viscosity on the bubble implosion, shockwave propagation, and shockwave impact onto bulk solid materials. Here, we used the X-ray imaging data to validate our model. Fig. 3a shows the dynamics of a single bubble in DIW and 50 cSt silicone oil, respectively, alongside the corresponding simulation results in Fig. 3b for comparison. The simulations show good agreement with the experimental observations.

### 3.1 Bubble dynamics in fluids of different viscosities

**Fig. 4:**
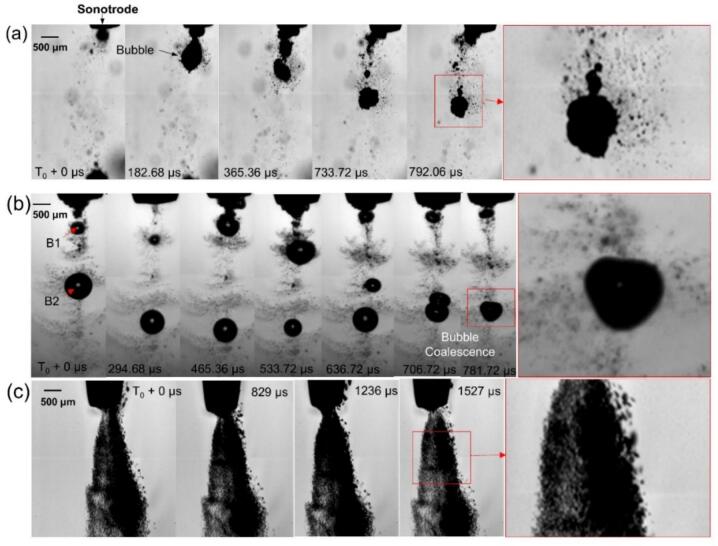
Three typical image sequences, showing the bubble dynamic behaviours in (a) DIW, (b) 50 cSt silicone oil and (c) 1000 cSt silicone oil below the sonotrode tip (11,000 fps). (More dynamic information is illustrated in Videos 2–4).

Ultrasound processing may produce significant changes on the viscosity of the processed liquids [51]. These changes are caused by numerous reasons, for example rising temperature and structure changes of the processed liquid, flow or shear induced thinning effect, etc. On the other hand, viscosity also has profoundly impact on bubble nucleation, growth, implosion, and shock wave propagation [52]. To further quantify and elucidate the effects of viscosity, we selected three representative fluids: DIW, low-viscosity silicone oil (50 cSt), and high-viscosity silicone oil (1000 cSt). Fig. 4(a–c) show some typical optical images, showing the dynamic behaviours of ultrasonic cavitation bubbles in DIW, 50 cSt, and 1000 cSt silicone oil, respectively. In DIW, cavitation bubbles can nucleate below the sonotrode tip “easily”, then grew rapidly and moved downward in the pressure field (Fig. 4a). In contrast, the bubbles in 50 cSt silicone oil (Fig. 4b) exhibit a more spherical shape because of the higher viscosity (more clearly by comparing the bubble morphology in the accompanying videos). In 1000 cSt oil (Fig. 4c), strong viscous damping effect limits bubble growth, resulting in tiny (∼2 μm) bubbles that stayed close together and moved down slowly.

The Reynolds number (Re=ρuRμ, u is the velocity of the bubble wall) is the ratio of the inertial force versus the viscous one. Assuming a bubble radius of 50  µm and a wall velocity of 0.21  m/s, as estimated from the X-ray imaging data, the Re number is calculated to be 10.5 in water and 0.0105 in 1000 cSt silicone oil. Hence, the inertial force dominates in DIW, causing higher degree of surface instabilities which lead to more distorted or deformed bubble morphology in DIW (see Fig. 3a). In contrast, silicone oil with higher viscosity results in a lower Re, and therefore the viscous force is “in control”, suppressing the velocity-induced disturbances at the bubble wall, making the bubble to maintain a more stable and spherical shape (see Fig. 3b). Similar phenomenon was reported by Rosselló *et al.* [53] when studied the bubble dynamics in water droplets, and found that the gas–water interface has higher degree of instabilities. As comparison, Li *et al.* [54] used laser to induce cavitation bubbles inside oil droplets and found that the viscous oil significantly suppressed oscillation-induced instability.

**Fig. 5:**
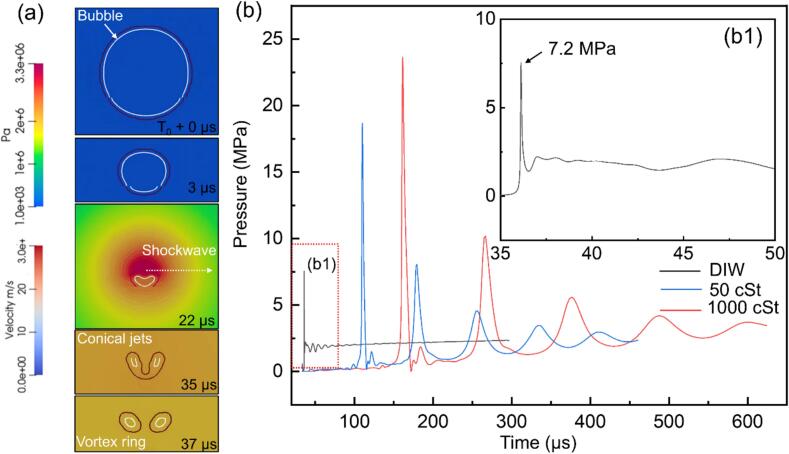
(a) Simulated sequences of individual bubble compression by acoustic pressure followed by implosion within one acoustic cycle in DIW; (b) the pressure profiles as a function of time for different viscosity media during bubble implosion. (More dynamic information is illustrated in Video 5).

**Fig. 6:**
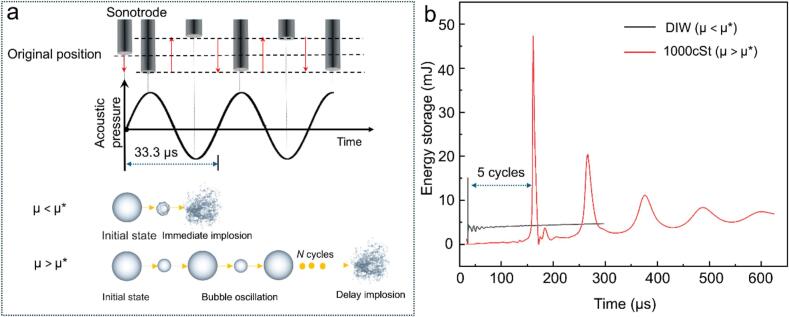
(a) Two representative cases of bubble dynamics above and below the critical viscosity threshold. When the viscosity is below the threshold, the bubble undergoes rapid implosion accompanied by relatively weaker shock emission. In contrast, when the viscosity exceeds the threshold, bubble implosion is delayed because much longer time is needed to accumulate the required energy for implosion to occur in a high-viscous fluid; (b) The stored energy profiles for the two cases, highlighting the difference in the energy retention in the high-viscous liquid.

Using this validated bubble model, we studied systematically the effects of viscosity on bubble dynamics. We used an initial acoustic pressure of 7.25 MPa, ensuring the occurrence of implosion of the bubble in silicone oils. Fig. 5a shows the simulation results of a single bubble and the corresponding pressure distribution over one acoustic cycle. The evolution of the bubble morphology is presented alongside pressure contour maps. Between 3 μs and 22 μs, the bubble undergoes intense compression, generating outward-propagating shock waves. By 35 μs, it develops into a distorted conical jet. Following jet penetration, the bubble evolves into a vortex ring (or toroidal) structure at 37 μs. The observed bubble dynamic behaviour agrees well with some previous simulation and experimental studies [55,56]. Fig. 5b shows the pressure wave propagation as a function of time for different viscosity cases. The 50 cSt Si oil produced a peak pressure of ∼ 17 MPa. The 1000 cSt one resulted in ∼ 24 MPa. Interestingly, secondary and even tertiary shockwaves occurred in the two silicone oil cases with reduced intensities (ranging from 4 to 8 MPa) due to the effect of viscous dissipation. As shown in Fig. 6a, there existed a critical viscosity, μ*, when a liquid of μ < μ*, the damping effect dominates, dissipating kinetic energy during bubble collapse. Increasing viscosity in this regime inhibits rapid bubble contraction, reduces minimum radius, and thereby reduce the intensity of the emitted shock wave. This behavior agrees with classical damping theory and is consistent with the experimental results reported by Luo *et al.*[57]. When μ > μ*: the system enters a high-damping regime, where the influence of viscosity becomes nonlinear. The bubble’s implosion and oscillation rates are significantly delayed due to increased viscous resistance. However, if the external driving forces (e.g., acoustic pressure) persist, internal pressure within the bubble gradually accumulates over time. This prolonged energy accumulation can eventually trigger a delayed bubble collapse, leading to a sudden release of energy, producing a shock wave with even higher intensity. Below, we estimate the viscosity threshold based on the energy conservation law.

The inertial energy of an imploding bubble:

(25) $$E_{\mathrm{inertia}}=\;\frac{1}{2}{\dot{R}}_{\max}^{2}$$

The viscous dissipation during the implosion time τ:

(26) $$E_{\mathrm{viscous}}\;=\frac{4\mu {\dot{R}}_{\max}^{2}}{\rho R_{\min}^{2}}∙\tau$$

Setting $E_{\mathrm{inertia}}=E_{\mathrm{viscous}}$, we obtain:

(27) $$\mu^{*}\approx \frac{\rho R_{\min}^{2}}{8\tau }$$

Using the representative parameters from our X-ray imaging work: initial radius: $R_{0}=300\;\mu m,$ minimum radius during implosion: $R_{\min}=30\;\mu m,$ driving frequency: f=30kHz, liquid density: $\rho =1000\;\mathrm{kg}/m^{3}$.

Substituting into Eq. (27), we obtain:$\mu^{*}\approx 13.7\;\mathrm{mPa}.s$. In our simulations, the silicone oils have viscosities of 50 mPa·s and 1000 mPa·s, both substantially exceeding the estimated threshold μ*. Consequently, the shock wave intensity was observed to increase with viscosity. Yasui *et al.* [58] also simulated the shock waves produced in viscous liquids. They showed that in a high viscous liquid (∼200 mPa·s) exposed to an acoustic pressure of ∼ 2 MPa, bubble implosions could be more violent than in pure water, which was consistent with our results here. These predictions have also been validated by experiments. For example, Zong *et al.* [59] reported the use of static pressure to assist large-scale ultrasonic exfoliation of graphite. Although higher static pressure suppresses the start of cavitation formation, it increased the violent behaviour at bubble implosion.

Formation of the intense secondary shockwaves in silicone oils is due to two factors: ***I. Temporary energy storage in viscous fluids:*** As the bubble implodes, its volume rapidly decreases, compressing both the internal gas and the surrounding liquid. The pressure-induced work during this collapse can be expressed as:

(28) W=∫PdV

where P is the internal gas pressure and dV is the change in bubble volume. In high-viscous fluids, internal friction slows the liquid’s movement, preventing rapid energy (shock wave) release. The corresponding profiles, as shown in Fig. 6b, reveal that in the high-viscous case, shock wave emission is delayed by nearly five cycles. Moreover, the peak energy stored reaches approximately 48  mJ, which is over three times higher than that in the low-viscous case (15  mJ).

II. ***Viscosity-induced lag:*** As the bubble reaches its minimum size, the internal gas pressure reached its peak. However, due to the lag induced by viscosity, the surrounding liquid continues to collapse inward, creating a transient imbalance between the rising internal pressure and the inward momentum of the liquid. This imbalance causes the bubble to rebound. The delayed response of the surrounding fluid leads to an expansion phase, during which a backflow wave forms and converges at the bubble’s center, releasing the stored energy and generating a short but intense secondary shockwave.

**Fig. 7:**
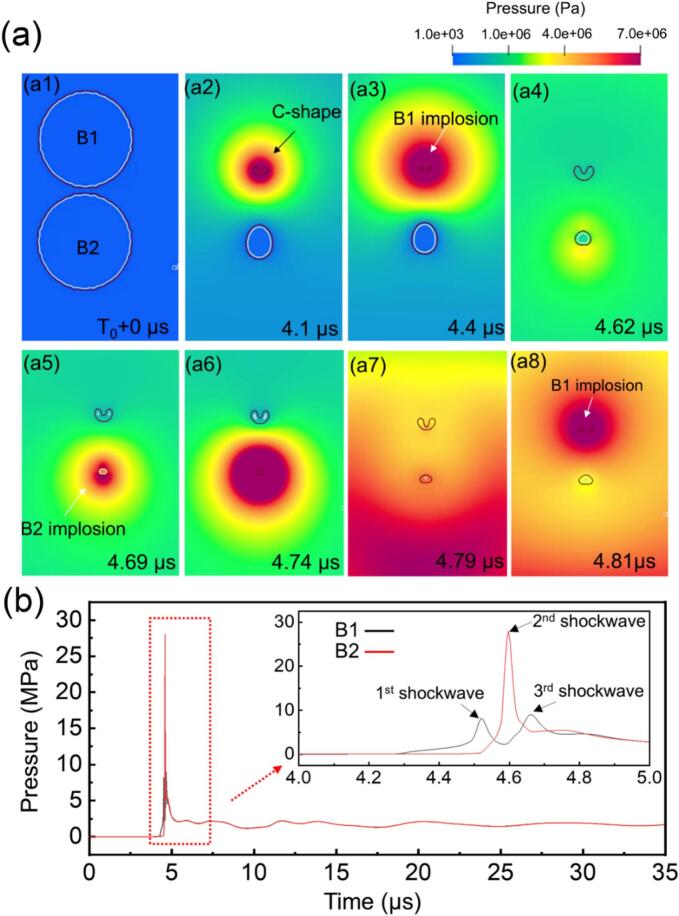
(a) The simulated sequence of interactions in a 2-bubble system in an ultrasound field, illustrating the chain reaction initiated by the implosion of one bubble; (b) the pressure profiles as a function of time in one ultrasound cycle at the center of bubbles B1 and B2. The inset in Fig. 7b shows an enlarged view of the red dotted region. (More dynamic information is illustrated in Video 6).

A comprehensive understanding of multiple bubble interactions is essential, as these interactions are pervasive and significantly influence the dynamics within cavitation zones, particularly as bubbles move away from the sonotrode tip, where its behavior becomes more intertwined. Here, we further investigate a two-bubble system, focusing specifically on how the implosion of one bubble to influence the other. Fig. 7a shows a simulation case in DIW (bubble B1 and B2). The sonotrode tip (initial pressure boundary) was 1 mm above B1. The simulation shows that B1 was rapidly compressed, formed a C-shape at 0.5 μs (Fig. 7a2) and then a shockwave emitted. The implosion of B1 subsequently triggered the implosion of B2 in 0.2 μs. At 6.8 μs, another shockwave was emitted by B2 and induced the secondary implosion of B1. Fig. 7b shows the shockwave pressure profiles as a function of time at the center of B1 and B2. Three implosion events can be clearly identified, occurred in a period of 0.5 μs. Notably, the shockwave produced by B2 reached a peak value of ∼ 27 MPa, significantly exceeding that by the 1st implosion of B1. Interestingly, with the same initial pressure input, no bubble implosion was seen in the two silicone oil cases.

**Fig. 8:**
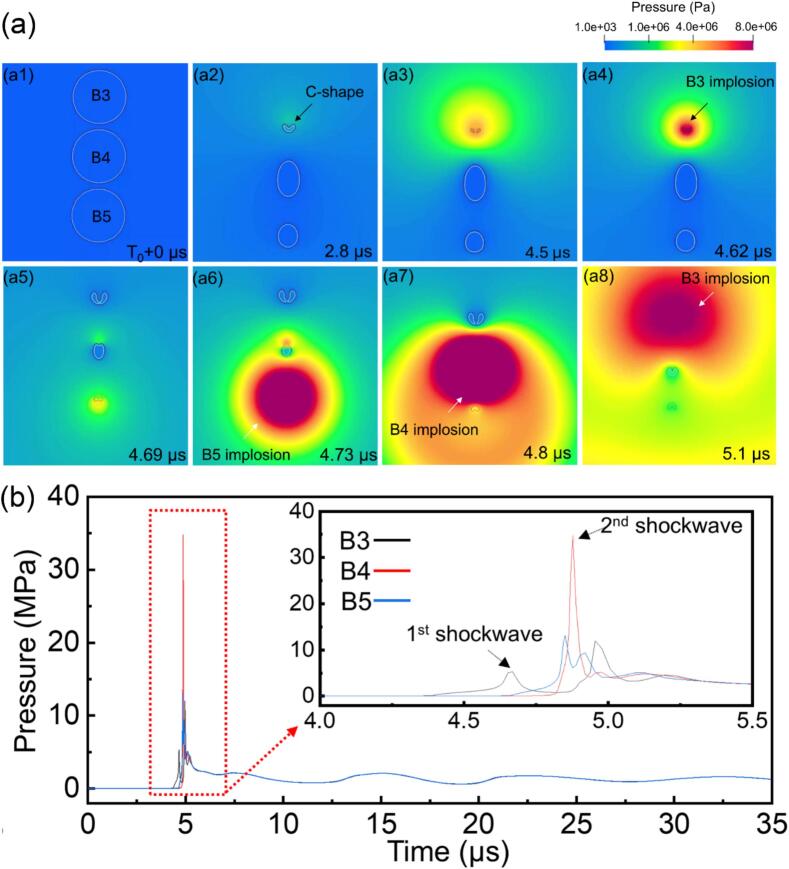
(a) The simulated sequence of interactions in a 3-bubble system in an ultrasound field, showing the chain reaction triggered by the initial implosion of B3; (b) the pressure profiles as a function of time for one cycle at the center of bubbles B3, B4 and B5. The inset in Fig. 8b shows an enlarged view of the red dotted region. (More dynamic information is illustrated in Video 7).

The cyclic acoustic pressure fields can trigger the nucleation of large number of bubbles at the same time, their collective growth often leads to the formation of a bubble cloud. To perform a tractable simulation for a bubble cloud, we did simulation of a 3-bubble system, studying particularly how one bubble implosion to create a chain reaction of implosion to others. Fig. 8a shows the initial bubble arrangement (again the input pressure boundary was 1 mm above bubble B3, see Fig. 8a1). After B3 imploded (see Fig. 8a5 ∼ 6), the produced shock wave propagated towards B4 and B5, triggering the implosion of B5 and then B4 with the time interval of just 0.07 μs. Fig. 8b presents the pressure profiles as a function of time at the center of B3, B4, and B5. The shockwaves emitted by B4 reached a peak value of ∼ 34 MPa.

If there is no interaction (interference of other bubbles or objects), a single bubble would implode after undergoing several cycles of oscillation. Simulations show that the chain reaction could significantly increase the frequency of bubble implosion. When the shockwave peak induced by bubble implosion reached the vicinity of the surrounding bubbles, the pressure gradient at the bubble interface increased sharply. This sudden increase forced the surrounding bubbles to implosion rapidly on a timescale (∼0.5 μs) much shorter than the acoustic pressure cycle (∼33.3 μs). This rapid implosion disrupted the original slow evolution, which is normally dominated by the pressure differential between the bubble interior and the surrounding fluids, leading to premature implosion. As the local pressure continues to rise, the acceleration of the bubble wall increased markedly, the radius decreased rapidly, and the internal gas was compressed nearly isentropically to extreme temperature and pressure. This leads to the release of more intense energy and stronger shockwaves, further amplifying the local impact onto the surrounding fluids.

The chain reaction revealed by the simulation can also be validated by the Rayleigh–Plesset theory [60]. We consider the phenomenon in DIW, assuming a liquid density of 1000 kg/m^3^, an initial maximum bubble radius of 10 μm, and a surface tension of 0.072 N/m. The relationship between the peak pressure generated by bubble implosion and the collapse time can be approximately expressed as:

(29) $$P_{\mathrm{peak}}=\;\rho {\left(\frac{R_{\max}}{t_{c}}\right)}^{2}$$

Where, $P_{\mathrm{peak}}$ is peak pressure generated by bubble implosion, ρ is liquid density, $t_{c}$ is implosion time, $R_{\max}$ is the initial maximum bubble radius. Since the simplified model mentioned above neglects factors such as liquid viscosity, compressibility, and deviations from ideal spherical collapse. The estimated pressure reaches as high as 127 MPa—significantly overestimating the value compared to the numerical model. In addition, the occurrence of this chain reaction is highly sensitive to the liquid’s viscosity. In silicone oil, such a reaction is less likely to occur because of the higher threshold required for bubble implosion and the greater dissipation of shockwaves.

### 3.2 Shockwave propagation in fluids with varying viscosities

**Fig. 9:**
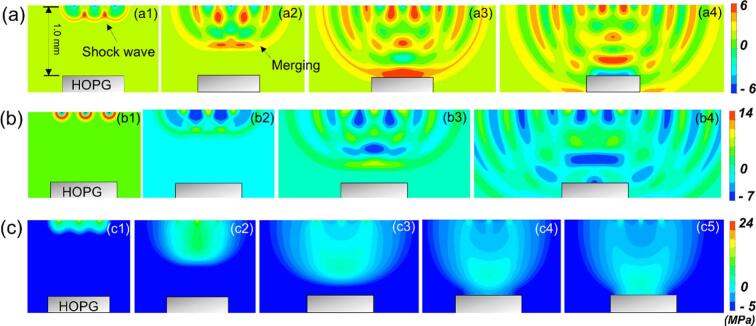
The simulated shockwave propagation sequences in fluids with different viscosities: (a) DIW (1 cSt); (b) silicone oil (50 cSt); (c) silicone oil (1000 cSt).

When multiple bubbles undergo implosion, each generates a rapid pressure buildup that evolves into a shockwave. The shockwaves from neighbouring bubbles interact and overlap, resulting in a cumulative effect where the individual shockwaves combine, thereby intensifying the overall pressure field. Here, we examine the dynamics of shockwave propagation in fluids with different viscosities. To enable a tractable simulation, we assume that the shockwaves generated by the three bubbles originate at the same vertical position along a horizontal line. We used the peak pressure of shock waves generated by bubble implosion in liquids of different viscosities (see Fig. 5) as the initial condition, i.e., 7.2 MPa in DIW, 17.2 MPa in 50 cSt silicone oil, and 24 MPa in 1000 cSt silicone oil. Fig. 9a1 illustrates the initial positions of these shockwave fronts.

Fig. 9(a–c) illustrate the propagation and interference behaviors of three shockwaves in fluids with varying viscosities. In the case of DIW, as the shockwaves propagated, the three individual fronts gradually merged into a single, larger wave that continued traveling downward. The interference between the shockwaves led to complex, unevenly distributed pressure contours, with alternating positive and negative pressure bands during propagation. Upon reaching the HOPG surface, the intensity of the shockwave remained nearly unchanged from the initial value (∼6 MPa), indicating minimal dissipation over a propagation distance of 1 mm. Although the waveform in 50 cSt silicone oil was similar to that in DIW (∼1 cSt), its wave intensity decreased from an initial 17 MPa to 7 MPa. Such attenuation is attributed to the higher viscosity that damps the wave propagation, resulting in higher energy dissipation. Interestingly, in 1000 cSt silicone oil, the earlier diffraction phenomenon disappears, and the wave characteristics are no longer obvious, indicating that at such high viscosity, the shock wave experiences significant damping. Despite this, the shock wave released by bubble implosion, which can reach up to ∼ 24 MPa, results in a wave intensity at the HOPG surface of approximately 9.7 MPa, still higher than that results in DIW.

### 3.3 Shockwaves impact onto the bulk materials

In our study here, the HOPG has hydrophobic surface [61], which promoted cavitation nucleation as argued by Belova *et al.* [62] and also confirmed by our in-situ X-ray imaging observations and simulation work [37]. In this work, we mainly focused on quantifying the shockwaves produced by the imploded bubbles above the HOPG surface and the subsequent impacts onto the HOPG surface.

**Fig. 10:**
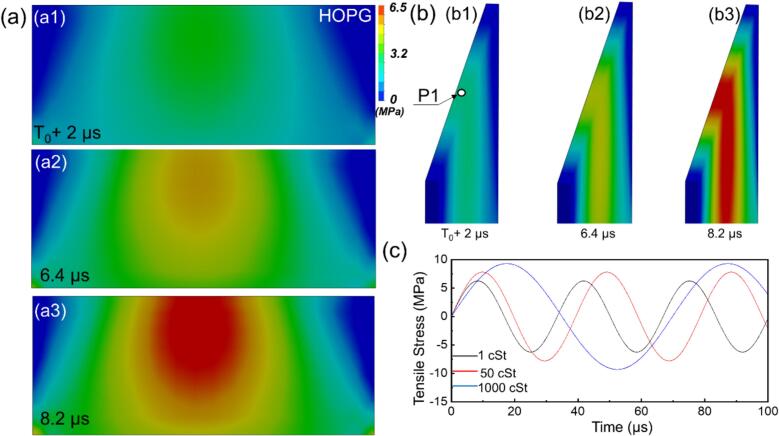
The simulated sequence of stress induced by shockwaves on to HOPG: (a) front view, (b) side view, and (c) stress profiles at point P1 of HOPG in fluids with viscosities of 1 cSt, 50 cSt, and 1000 cSt.

The WSI model allows us to calculate the stress produced in the HOPG induced by the shockwaves. Fig. 10a and b show the stress distribution across the HOPG, where the central region experienced the highest stress, reaching up to ∼ 6.5 MPa in water. A significant gradient was observed across the cross-section, with stress levels near the corners approaching zero. To further quantify these effects, Fig. 10c presents the tensile stress profiles at point P1 (center of HOPG) in the fluids with different viscosities.

Despite the significantly higher shock wave energy dissipation in viscous liquids such as 50 cSt and 1000 cSt silicone oils compared to DIW, the tensile stress transmitted to the HOPG surface is slightly greater, increasing from approximately 6.5  MPa in DIW to around 10  MPa in 1000 cSt silicone oil. It is clearly evident that viscosity significantly increases the threshold for cavitation bubble implosion and inhibits shockwave propagation [63]. Several studies [52] have also demonstrated that when the ultrasonic exfoliation or dispersion reached 180 mins, the viscosity of the dispersion liquid could rise as high as 3200 cSt. Our findings suggest that effective dispersion or exfoliation remains achievable even under highly viscous conditions, if bubble implosion can still occur when the acoustic pressure exceeds the threshold. Moreover, the selection of an appropriate ultrasound frequency is critical; lower frequencies may be necessary to induce effective cavitation in high-viscosity liquid.

## 4 Conclusion

We have developed a mathematical and numerical modelling framework for simulations of the complex physics and highly dynamic phenomena in the processes of sonochemistry and sonication of materials. We also did systematic model validation and calibration using the *in-situ* and real-time collected big X-ray image data. The key findings of this research are:

1. Viscosity has different effects. At the bubble growth stage, the damping effect can suppress bubble oscillations and therefore reducing interfacial instabilities. For bubble implosion, there exists a critical viscosity threshold. Below the threshold, bubble implosion may occur quickly but with less intensive shock wave due to the damping effect. Above the threshold, sufficient time is needed to accumulate energy for bubble implosion to occur (need longer time for incubation), but once imploded, it produces much intensive shock waves which may overcome the viscous damping effect.
2. For a system containing multiple bubbles, the shockwave generated by the implosion of one bubble can trigger chain implosion actions of other bubbles. In the chain implosion, multiple shockwaves can be triggered in a few tens of nanoseconds and peak pressure could be much higher than those triggered only by the original ultrasound pressure fields.
3. In addition to the most advanced *in-situ* and operando experimental approaches, this integrated model is an indispensable modelling tool for computational studies and optimizations of the ultrasound-assisted chemical synthesis and sonoprocessing of materials.

## CRediT authorship contribution statement

**Ling Qin:** Writing – original draft, Visualization, Validation, Software, Methodology, Formal analysis, Conceptualization. **Kang Xiang:** Writing – review & editing, Investigation. **Lianxia Li:** Writing – review & editing, Visualization, Software, Methodology, Investigation. **Iakovos Tzanakis:** Writing – review & editing, Funding acquisition. **Dmitry Eskin:** Writing – review & editing, Funding acquisition. **Jiawei Mi:** Writing – review & editing, Supervision, Resources, Project administration, Methodology, Funding acquisition.

## Declaration of competing interest

The authors declare that they have no known competing financial interests or personal relationships that could have appeared to influence the work reported in this paper.
